# Blood Pressure Control and Prescription Pattern of Antihypertensive Drugs in Adherence to the 2020 International Society of Hypertension (ISH) Global Hypertension Practice Guidelines in Saudi Arabia: A Retrospective Study

**DOI:** 10.7759/cureus.34965

**Published:** 2023-02-14

**Authors:** Mohammad A Rashikh, Abdulmgeed F Alruways, Hallal B Alotaibi, Nemer A Alotaibi, Feras Almarshad, Saad M Alsaab, Ghallab Alotaibi

**Affiliations:** 1 Department of Pharmacology, College of Medicine, Shaqra University, Dawadmi, SAU; 2 College of Medicine, Shaqra University, Dawadmi, SAU; 3 Department of Pediatrics, Dawadmi College of Medicine, Shaqra University, Riyadh, SAU; 4 Department of Internal Medicine, College of Medicine, Shaqra University, Shaqra, SAU; 5 Department of Internal Medicine, College of Medicine, Shaqra University, Dawadmi, SAU; 6 Department of Pharmacology, College of Pharmacy, Shaqra University, Dawadmi, SAU

**Keywords:** congestive heart failure, chronic kidney diseases, stroke, ischemic heart disease, irrational prescribing, elderly, diabetes mellitus

## Abstract

Background

Hypertension is the leading risk factor for cardiovascular disease and death. Appropriate treatment of hypertension is necessary to reduce mortality. A prescription-based study is one of the most influential and helpful methods to examine physicians’ irrational prescribing practices. This study was designed to investigate the antihypertensive prescription of physicians and their adherence to the treatment guidelines, as well as the blood pressure (BP) control rate in a general hospital in the Kingdom of Saudi Arabia.

Methodology

A retrospective, cross-sectional study was conducted between February 2020 and June 2021 in an outpatient department. Patients diagnosed with hypertension as per the 2020 International Society of Hypertension guidelines and those who received antihypertensive drugs were included. Study data included prescriptions, patient’s age, duration of hypertension, comorbidities, BP, drug therapy type, and antihypertensive class.

Results

Overall, angiotensin-converting enzyme inhibitors/angiotensin II receptor blockers (67.1%) were the most prescribed agents, followed by dihydropyridine-calcium channel blockers (62.6%), diuretics (26.1%), and β-blockers (10.1%). Comorbid and stage 2 hypertensive patients mainly received combination therapy (51.6%) rather than monotherapy (48.4%). The study revealed an 83.5% prescription adherence to the treatment guidelines. However, non-adherence was encountered in monotherapy, polytherapy, and elderly-treated patient groups. A 66.4% (at target BP in all cases <140/90 mmHg) and 39.3% (at target BP in comorbid patients <130/80 mmHg) rate of BP control was observed. Furthermore, the rate of BP control was significantly associated with prescription adherence (χ^2^ = 71.316; p < 0.001).

Conclusions

The degree of prescription adherence and rate of BP control were found to be compatible with other published hypertension studies. However, considerable scope exists for improvement in rational drug utilization and rate of BP control, particularly in high-risk patients. Therefore, treatment guidelines must be followed by clinicians to achieve BP goals and reduce cardiovascular events among the Saudi population.

## Introduction

Hypertension is one of the major preventable risk factors for heart disease and stroke [1]. Hypertensive patients constitute approximately 24% of total deaths from cardiovascular disease (CVD) [2]. According to the current data, more than one billion adults are diagnosed with hypertension worldwide, which is expected to reach 1.56 billion by 2025 [2]. The prevalence of hypertension (15.2%) and borderline hypertension (46.6%) is very high in Saudi Arabia [3]. Several studies have shown poor blood pressure (BP) control in Saudi Arabia [3,4] and other countries [5,6].

The rational use of antihypertensive drugs is one of the leading global challenges. Studies have revealed that irrational prescribing practices are associated with increased morbidity and mortality [5-7]. Therefore, appropriate screening, treatment, and adherence to hypertension prescription are necessary to reduce morbidity and mortality. A prescription for hypertension treatment is complex and depends on many factors, such as age, polypharmacy, comorbidities, duration of hypertension, and non-compliance [5-7]. Several guidelines have been established to prevent hypertension complications and improve the life span [8-10]. Prescription adherence to the 2020 International Society of Hypertension (ISH) Global Hypertension Practice Guidelines [8], European Society of Cardiology or European Society of Hypertension (ESC/ESH) [9], and 2018 Saudi Hypertension Guidelines [10] are highly recommended to prevent morbidity and mortality. Physicians must follow these global guidelines to manage hypertension and adhere to rational prescription practices. According to the 2020 ISH Global Hypertension Practice Guidelines, hypertension refers to a systolic/diastolic BP reading ≥140/90 mmHg [8]. Calcium channel blockers (CCBs), thiazide (TZD)/TZD-like diuretics, angiotensin-converting enzyme inhibitors (ACEIs), and angiotensin receptor blockers (ARBs) are recommended as first-line agents to treat hypertension [8,9]. However, β-blockers are no longer considered first-line drugs unless patients have ischemic heart disease (IHD), heart failure (HF), or atrial fibrillation (AF) and are younger women who are pregnant or planning a pregnancy [8,9].

The prescribing patterns of antihypertensive medication studies are beneficial in monitoring and evaluating prescribing habits to achieve better outcomes and patient safety [11-13]. Studies have demonstrated that poor adherence to the therapeutic plan of antihypertensive medication is correlated with increased CVD events [12,14]. From this perspective, this study was designed to evaluate the prescription pattern of antihypertensive drugs focusing on adherence and BP control.

## Materials and methods

Study design and participants

We conducted a retrospective, cross-sectional study among established hypertensive patients. The study was conducted between February 2020 and June 2021 in the medicine outpatient department (OPD) at Dawadmi General Hospital, Riyadh, Saudi Arabia. The inclusion criteria required that the patient had a clinic systolic/diastolic BP (SBP/DBP) ≥140/90 mmHg at the time of diagnosis, had received at least one antihypertensive medication, had Saudi citizenship, and was aged ≥18 years. The study excluded pregnant women.

Sample size

The sample size was calculated using the following formula described by Alruways et al. [15] based on the prevalence of hypertension of 15.2%, as reported in a previous local study [4], at a 95% confidence interval with a 5% allowable error: n = z^2^ pq/I^2^, where n is the minimum sample size; z is 1.96, which corresponds to 95% confidence interval; p is the proportion of the study population estimated to have hypertension (15.2%) (p = 0.152); q is 1.0 - p = 1.0 - 0.152 = 0.848; I is the allowable error (5%) = 0.05. Hence, the sample size was calculated as n = (1.96)^2^ × 0.152 × 0.848/(0.05)^2^ = 199. This formula gave the most diminutive sample size of 199, which was exceeded by enrolling 417 participants.

Blood pressure measurement

The office BP method was used to diagnose hypertension. Two readings (at one-minute intervals) of BP levels were taken and averaged on at least two occasions [8].

Criteria of non-adherence to antihypertensive therapy

Healthcare practitioners define irrational therapy as not following the treatment guidelines. The established criteria in defining irrational therapy are (a) monotherapy prescribed to grade 2 hypertensive patients; (b) monotherapy prescribed to high-risk grade 1 hypertensive patients aged less than 80 years; (c) β-blockers used without complaints of IHD, HF, AF, or pregnancy; (d) triple therapy prescribed without TZD/TZD-like diuretics; (e) ACEI and ARB concurrently used; (f) ACEI/ARB monotherapy prescribed to elderly patients (age ≥75 years) and grade 1 hypertensive patients; (g) polytherapy prescribed to elderly patients (age ≥80 years) and low-risk grade 1 hypertensive patient; and (h) short-acting DHP-CCB (nifedipine) prescribed for IHD [8-10].

Data collection

We reviewed 417 outpatient prescriptions from patients diagnosed with hypertension and prescribed at least one antihypertensive drug. Data collected from physicians’ prescribing records of medicine OPD of Dawadmi General Hospital included the duration of hypertension, SBP, DBP, and comorbidities such as diabetes mellitus (DM), dyslipidemia (DLP), IHD, chronic kidney disease (CKD), congestive heart failure (CHF), and stroke. Before data collection, we obtained consent from the Dawadmi General Hospital Authority and the Ministry of Health, Saudi Arabia. All data were kept confidential during and after the study.

Statistical analysis

Data were analyzed using SPSS version 25 (IBM Corp., Armonk, NY, USA). A one-sample binomial or chi-square (χ^2^) test was applied to examine the statistical significance. Continuous variables were presented as the mean ± standard deviation (SD), and categorical variables were presented as frequencies and percentages. All comparisons were considered significant at p-values <0.05.

Ethical approval

Ethical approval was obtained from the Central Institutional Review Board, Ministry of Health, Saudi Arabia (central IRB log number: 20-09E) and Medical Research Ethics Committee, College of Medicine, Dawadmi, Shaqra University, Saudi Arabia (project number: CMD/DWD/SU/2019/11/004).

Ethics guideline statement

The 2020 ISH Global Hypertension Practice Guidelines [8] and the 2018 Saudi Hypertension Guidelines [10] were used to evaluate prescription adherence to antihypertensive therapy.

## Results

Characteristics of the study population

In total, 417 prescriptions fulfilled the inclusion criteria. Of the total, 78.2% of the patients were under 65 years of age. The average age of the study population was 56.58 ± 11.11 years. Our study found that more than half (64.7%) of the participants had at least one comorbidity. Regarding comorbidities, most hypertensive patients had DM (48.2%), followed by DLP (16.3%), IHD (5.3%), stroke (3.1%), CKD (2.9%), and CHF (1.9%), as summarized in Table 1.

**Table 1:** Characteristics of the study population (n = 417). n = number of patients; % = percentage; BP = blood pressure; SBP = systolic blood pressure; DBP = diastolic blood pressure

Variables		n (%)
Age (years), mean ± SD = 56.58 ± 11.11	18–64	326 (78.2)
	≥65	91 (21.8)
No comorbidity		147 (35.3)
Total comorbid patients		270 (64.7)
Diabetes mellitus		201 (48.2)
Dyslipidemia		68 (16.3)
Ischemic heart disease		22 (5.3)
Stroke		13 (3.1)
Chronic kidney disease		12 (2.9)
Congestive heart failure		8 (1.9)
Controlled BP (SBP/DBP <140/90 mmHg)		277 (66.4)
Uncontrolled BP (SBP/DBP ≥140/90 mmHg)		140 (33.6)

Table 2 displays the stages of hypertensive patients according to the 2020 ISH Global Hypertension Practice Guidelines. Regarding SBP, 27.1%, 30.7%, 31.6%, and 10.5% of patients were classified as normal, high normal, grade I, and grade II, respectively. Regarding DBP, 57.5%, 25.2%, 10.5%, and 6.7% of patients were classified as normal, high normal, grade I, and grade II, respectively.

**Table 2:** Classification of hypertensive patients based on the 2020 ISH Global Hypertension Practice Guidelines (n = 417). n = number of patients; % = percentage; BP = blood Pressure; ISH = International Society of Hypertension

Systolic blood pressure	n (%)	Diastolic blood pressure	n (%)
Normal BP (<130 mmHg)	113 (27.1)	Normal BP (<85 mmHg)	240 (57.5)
High-normal BP (130–139 mmHg)	128 (30.7)	High-normal BP (85–89 mmHg)	105 (25.2)
Grade 1 hypertension (140–159 mmHg)	132 (31.6)	Grade 1 hypertension (90–99 mmHg)	44 (10.5)
Grade 2 hypertension (≥160 mmHg)	44 (10.5)	Grade 2 hypertension (≥100 mmHg)	28 (6.7)

Utilization of different classes of antihypertensive drugs

**Figure 1:**
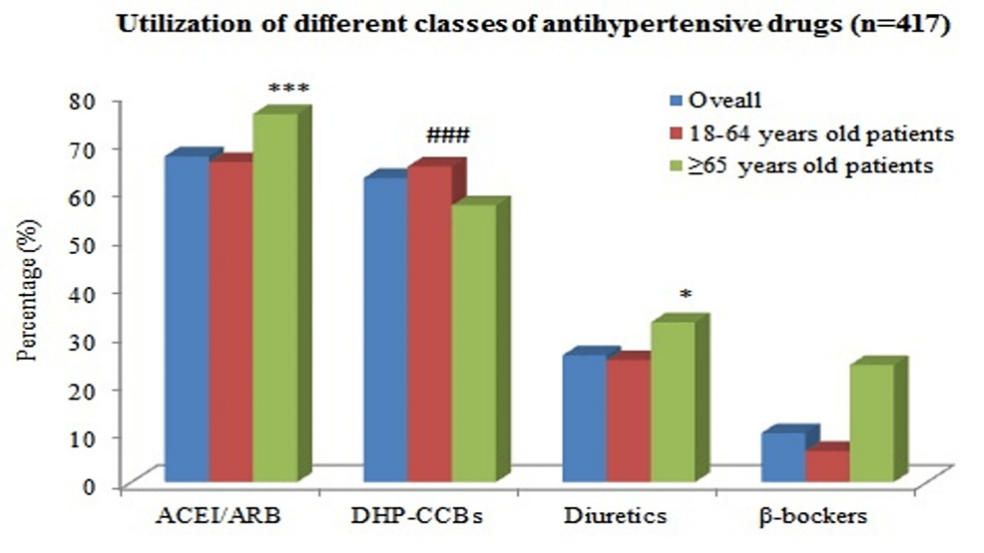
Utilization of different classes of antihypertensive drugs (n = 417). n = total number of prescriptions; DHP-CCB = dihydropyridine-calcium channel blocker; ACEI = angiotensin‑converting enzyme inhibitor; ARB = angiotensin receptor blocker One-sample binomial test was used for analysis; p < 0.05 was considered statistically significant. *: (p < 0.05) use of diuretics in young age group versus elderly age group. ***: (p < 0.001) use of ACEIs/ARBs in young age group versus elderly age group. ^###^: (p < 0.001) use of DHP-CCBs in young age group versus elderly age group.

Figure 1 describes the overall utilization of antihypertensive drugs. ACEIs/ARBs (67.1%) were the most used antihypertensive drugs, followed by DHP-CCBs (62.6%), diuretics (26.1%), and β-blockers (10.1%). Regarding the age groups, the elderly group of hypertensive patients received a significantly higher percentage of ACEIs/ARBs and diuretics than the younger group (p < 0.05). However, the younger group of hypertensive patients received a higher percentage of DHP-CCBs compared to the elderly age group (p < 0.001).

Utilization of antihypertensive drugs as monotherapy or polytherapy in different age groups

**Figure 2:**
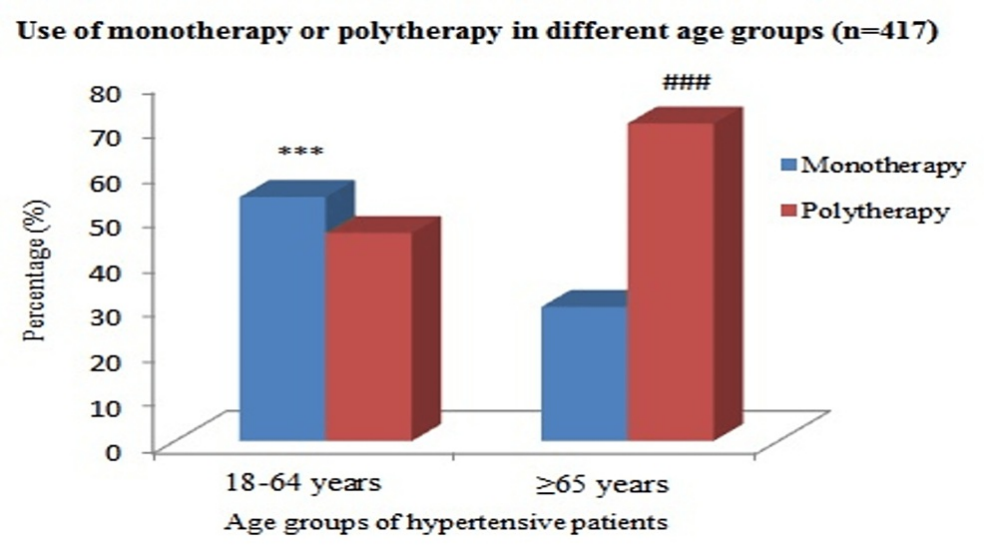
Use of antihypertensive drugs as monotherapy or polytherapy in different age groups. Pearson chi-square (χ^2^) test was used for analysis; p < 0.05 was considered statistically significant, and p < 0.001 was considered statistically highly significant. ***: (p < 0.001) use of monotherapy in younger age group versus elderly age group of hypertensive patients. ^###^: (p < 0.001) use of polytherapy in younger age group versus elderly age group of hypertensive patients.

Most younger age groups received monotherapy which is significantly higher compared to the older group of hypertensive patients (54% versus 29.7%; p < 0.001). However, polytherapy was more commonly used in the elderly group of hypertensive patients (70.3% versus 46%; p < 0.001), as summarized in Figure 2.

Utilization of antihypertensive drugs as monotherapy or polytherapy in comorbidity

**Figure 3:**
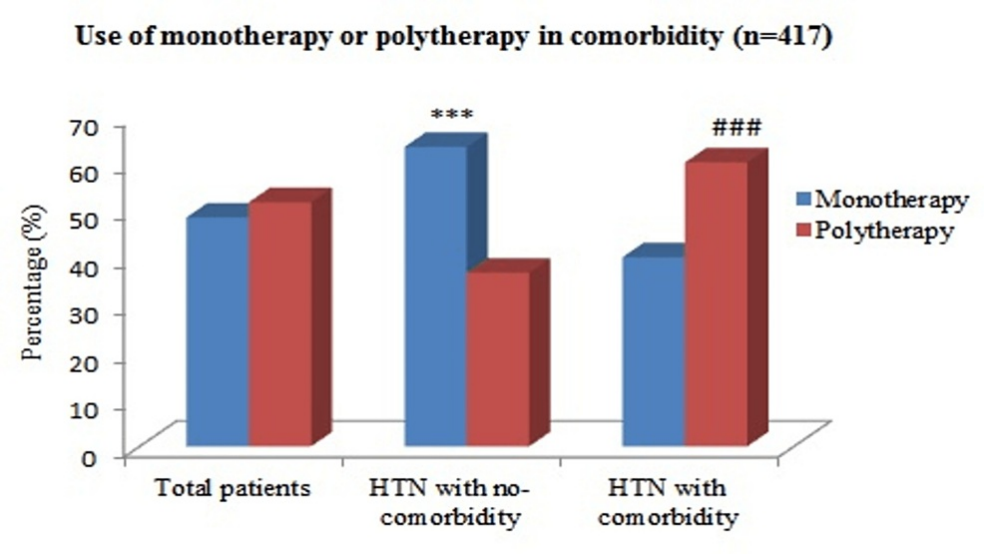
Use of antihypertensive drugs as monotherapy or polytherapy in comorbidity. HTN = hypertension Pearson chi-square (χ^2^) test was used for analysis; p < 0.05 was considered statistically significant, and p < 0.001 was considered statistically highly significant. ***: (p < 0.001) use of monotherapy in comorbid versus non-comorbid hypertensive patients. ^###^: (p < 0.001) use of polytherapy in comorbid versus non-comorbid hypertensive patients.

Figure 3 describes the number of antihypertensive drugs and prescription rates by concomitant disease. Polytherapy was prescribed significantly higher for comorbid hypertensive patients than for non-comorbid hypertensive patients (60% versus 35.4%; p < 0.001).

Types of antihypertensive therapy and duration of hypertension

**Figure 4:**
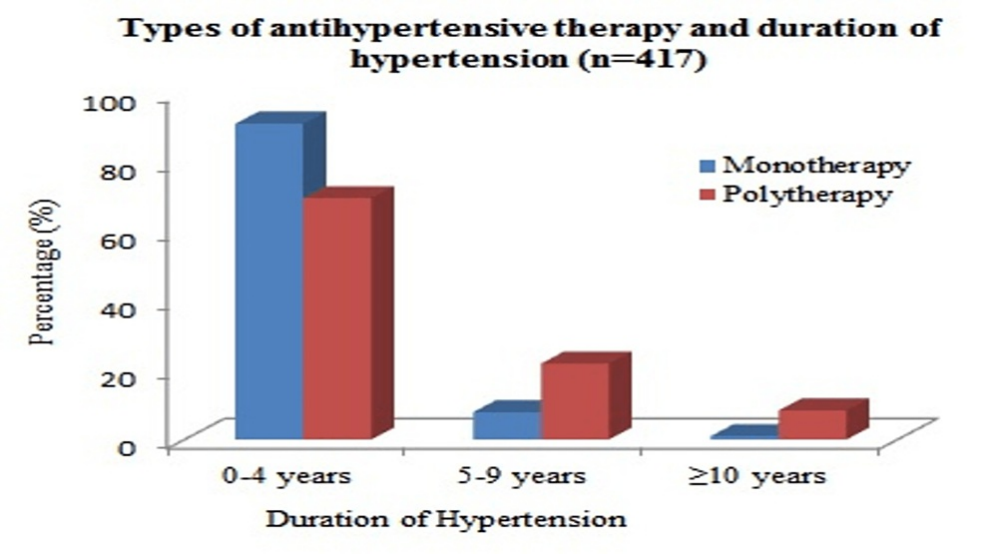
Types of antihypertensive therapy and duration of hypertension.

Utilization of polytherapy was found to be significantly higher among patients who had hypertension for the last five years or more than five years, as summarized in Figure 4.

Prescription pattern of antihypertensive drugs in hypertensive patients with or without comorbidity

The study results revealed that most hypertensive patients received combination therapy (51.6%) rather than monotherapy (48.4%). Overall, renin-angiotensin system (RAS) blockers (ACEIs/ARBs, 67.1%) were the most prescribed antihypertensive agents, followed by DHP-CCBs (62.6%), diuretics (26.1%), and β-blockers (10.1%). Hypertensive patients who had no comorbidity received mostly DHP-CCBs (71.4%), followed by RAS blockers (48.3%), diuretics (24.5%), and β-blockers (2.7%). The hypertensive patients who had DM mostly received RAS blockers (75.5%), followed by DHP-CCBs (59.2%), diuretics (21.4%), and β-blockers (6.5%). However, RAS blockers (83.3%) and diuretics (58.4%) were the most preferred choice among CKD patients. Polytherapy was mainly prescribed to DM (53.2%), DLP (58.8%), and CKD (91.7%) hypertensive patients, as summarized in Table 3.

**Table 3:** Prescription pattern of antihypertensive drugs in hypertensive patients with or without comorbidity. HTN = hypertension; n = number of prescriptions; DHP-CCB = dihydropyridine-calcium channel blocker; RAS = renin-angiotensin system; ACEI = angiotensin‑converting enzyme inhibitor; ARB = angiotensin receptor blocker; TZD = thiazide; DM = diabetes mellitus; DLP = dyslipidemia; CKD = chronic kidney disease

Classes of antihypertensive drugs		Overall (n = 417)	HTN with no comorbidity (n = 147)	HTN with DM (n = 201)	HTN with DLP (n = 68)	HTN with CKD (n = 12)
Use of different classes of antihypertensives as monotherapy; n (%)	RAS blockers (ACEI/ARB)	75 (18)	19 (12.9)	50 (24.8)	16 (23.5)	0
	DHP-CCB	126 (30.2)	74 (50.3)	44 (21.9)	12 (17.6)	1 (8.3)
	β-blockers	1 (0.2)	0	0	0	0
	Total monotherapy	202 (48.4)	93 (63.3)	94 (46.8)	28 (41.2)	1 (8.3)
Use of different classes of antihypertensives as polytherapy; n (%)	RAS blockers	205 (49.1)	52 (35.4)	102 (50.7)	38 (55.9)	10 (83.3)
	DHP-CCB	135 (32.4)	31 (21.1)	75 (37.3)	28 (41.2)	6 (50)
	TZD/TZD-like diuretic	95 (22.8)	36 (24.5)	43 (21.4)	13 (19.1)	2 (16.7)
	β-blockers	41 (9.8)	4 (2.7)	13 (6.5)	8 (11.7)	5 (41.7)
	Loop diuretics	14 (3.3)	0	0	0	5 (41.7)
	Potassium-sparing diuretics (spironolactone)	5 (1.2)	0	0	0	1 (8.3)
	α-blocker (tamsulosin)	1 (0.2)	0	0	0	0
	Dual therapy	153 (36.7)	40 (27.2)	87 (43.3)	33 (48.5)	5 (41.7)
	Triple therapy	55 (13.2)	12 (8.2)	20 (10)	7 (10.3)	4 (33.3)
	Four-drug therapy	7 (1.7)	2 (1.4)	0	0	2 (16.7)
	Total polytherapy	215 (51.6)	54 (36.7)	107 (53.2)	40 (58.8)	11 (91.7)

Use of antihypertensive drugs in hypertensive patients with ischemic heart disease, stroke, or congestive heart failure or in the elderly

Hypertensive patients who had IHD mostly received RAS blockers (86.3%), followed by β-blockers (72.7%), DHP-CCBs (40.9%), and diuretics (40.9%). However, RAS blockers (92.3%) and DHP-CCBs (69.2%) were the most preferred choice among stroke patients, followed by β-blockers (68.2%) and diuretics (38.4%). CHF patients received mainly diuretics (75%) and RAS blockers (75%), followed by DHP-CCBs (37.5%) and β-blockers (37.5%). However, elderly patients primarily received RAS blockers (73.6%), DHP-CCBs (69.2%), diuretics (31.9%), and β-blockers (24.2%). Regarding types of antihypertensive therapy, polytherapy was mainly used among the elderly (70.3%), IHD (81.8%), stroke (84.6%), and CHF (87.5%) hypertensive patients, as summarized in Table 4.

**Table 4:** Prescription pattern of antihypertensive drugs in hypertensive patients with IHD, stroke, or CHF or in the elderly. n = number of prescriptions; % = percentage; HTN = hypertension; DHP-CCB = dihydropyridine-calcium channel blocker; RAS = renin-angiotensin system; ACEI = angiotensin‑converting enzyme inhibitor; ARB = angiotensin receptor blocker; TZD = thiazide; IHD = ischemic heart disease; CHF = congestive heart failure

Classes of antihypertensive drugs		HTN + IHD patients (n = 22)	HTN + stroke patients (n = 13)	HTN + CHF patients (n = 8)	HTN in elderly patients (≥65 years) (n = 91)
Use of different classes of antihypertensives as monotherapy; n (%)	RAS blockers (ACEI/ARB)	1 (4.5)	2 (15.4)	0	7 (7.7)
	DHP-CCB	2 (9.1)	0	1 (12.5)	20 (22)
	β-blocker	1 (4.5)	0	0	0
	Total monotherapy	4 (18.2))	2 (15.4)	1 (12.5)	27 (29.7)
Use of different classes of antihypertensives as polytherapy; n (%)	RAS blockers	18 (81.8)	10 (76.9)	6 (75)	60 (65.9)
	DHP-CCB	7 (31.8)	9 (69.2)	2 (25)	43 (47.2)
	TZD/TZD-like diuretic	7 (31.8)	4 (30.7)	0	23 (25.3)
	β-blockers	15 (68.2)	0	3 (37.5)	22 (24.2)
	Loop diuretics	2 (9.1)	1 (7.7)	6 (75)	6 (6.6)
	Potassium-sparing diuretics (spironolactone)	0	1 (7.7)	3 (37.5)	0
	Dual therapy	7 (31.8)	8 (61.5)	2 (25)	44 (48.3)
	Triple therapy	9 (40.9)	3 (23.1)	4 (50)	14 (15.4)
	Four-drug therapy	2 (9.1)	0	1 (12.5)	6 (6.6)
	Total polytherapy	18 (81.8)	11 (84.6)	7 (87.5)	64 (70.3)

Non-adherence to antihypertensive treatment

The results indicated 17.7%, 10.7%, and 11% non-adherence prescriptions with monotherapy, polytherapy, and elderly treated patients, respectively. The non-adherence prescription finding was (a) 4.9% monotherapy prescribed to grade 2 hypertensive patients; (b) 12.2% monotherapy prescribed to high-risk grade 1 hypertensive patients aged less than 80 years; (c) 3.7% and 0.5% β-blockers used in dual and triple therapy, respectively, as first-line antihypertensive without IHD, HF, AF, or pregnancy; (d) 4.6% triple therapy prescribed without TZD/TZD-like diuretics; (e) ACEI plus ARB concurrently used (1.4%); (f) ACEI/ARB monotherapy (8.8%) prescribed to elderly patients (age ≥75 years) and grade 1 hypertensive patients; (g) 2.2% polytherapy prescribed to elderly patients (age ≥80 years) and low-risk grade 1 hypertensive patients; and (h) 0.5% monotherapy of short-acting DHP-CCB prescribed to IHD, as summarized in Tables 5-7.

**Table 5:** Non-adherence encountered with monotherapy of antihypertensive drugs (total prescriptions = 203). n = number of prescriptions; % = percentage; DHP-CCB = dihydropyridine-calcium channel blocker; ACEI = angiotensin‑converting enzyme inhibitor; ARB = angiotensin receptor blocker; IHD = ischemic heart disease; HTN = hypertension

Monotherapy	Overall, n (%)	Monotherapy in grade 2 hypertensive patients, n (%)	Monotherapy in high-risk hypertensive patients + grade 1 HTN + aged <80 years, n (%)	Use of short-acting DHP-CCBs in IHD patients, n (%)
DHP-CCBs	17 (8.3)	6 (2.9)	10 (4.9)	1 (0.5)
ACEI/ARBs	19 (9.3)	4 (1.9)	15 (7.3)	0
Total	36 (17.7)	10 (4.9)	25 (12.3)	1 (0.5)

**Table 6:** Non-adherence encountered with combination therapy of antihypertensive drugs (total prescriptions = 214). n = number of prescriptions; % = percentage; DHP-CCB = dihydropyridine-calcium channel blocker; ACEI = angiotensin‑converting enzyme inhibitor; ARB = angiotensin receptor blocker; TZD = thiazide; BB = β-blockers; IHD = ischemic heart disease; HF = heart failure; HTN = hypertension; edematous conditions = HF, chronic kidney disease, liver failure, glomerulopathy

	Overall	BB in dual therapy without IHD/HF	BB in triple therapy without IHD/HF	Triple therapy without TZD/TZD-like diuretics	Wrong association
DHP-CCB + BB	4 (1.8)	4 (1.8)	0	0	0
ACEI/ARB + BB	4 (1.8)	4 (1.8)	0	0	0
ACEI + ARB	1 (0.5)	0	0	0	1 (0.5)
ARB/ACEI + DHP-CCB + BB	9 (4.2)	0	0	9 (4.2)	0
ACEI + DHP-CCB + loop diuretics (without edematous conditions)	1 (0.5)	0	0	1 (0.5)	0
DHP-CCB + TZD diuretics + BB	1 (0.5)	0	1 (0.5)	0	0
Loop diuretic + aldosterone antagonist + BB	1 (0.5)	0	1 (0.5)	0	0
ACEI + ARB + TZD diuretics	1 (0.5)	0	0	0	1 (0.5)
ACEI + ARB + DHP-CCB + BB	1 (0.5)	0	0	0	1 (0.5)
Total	23 (10.7)	8 (3.7)	2 (0.5)	10 (4.6)	3 (1.4)

**Table 7:** Non-adherence encountered in elderly hypertensive treated patients (n = 91). n = number of prescriptions; % = percentage; ACEI = angiotensin‑converting enzyme inhibitor; ARB = angiotensin receptor blocker; HTN = hypertension

Types of HTN	Overall	Use of ACEI/ARBs as monotherapy in elderly patients (aged ≥75 years)	Use of polytherapy in very old patients (aged ≥80 years) with no comorbidity
Grade 1 HTN, n (%)	10 (11)	8 (8.8)	2 (2.2)

Association of blood pressure control with prescription adherence according to cut-off points

Among the total patients, the result demonstrated an 83.5% degree of prescription adherence and a 66.4% rate of BP control (at BP target ≤140/90 mmHg). Moreover, BP control was significantly associated with prescription adherence (χ^2^ = 71.316; p < 0.001). In hypertension with comorbidity groups, our study revealed a 78.5% degree of prescription adherence. However, the rate of BP control dropped to 39.3% at a lower cut-off point (target BP <130/80 mmHg) and exhibited a significant association with prescription adherence (χ^2^ = 11.784; p < 0.001), as summarized in Table 8.

**Table 8:** Association of BP control with prescription adherence according to cut-off points. n = number; % = percentage; BP = blood pressure; SBP = systolic blood pressure; DBP = diastolic blood pressure Chi‑square (χ^2^) test was used for analysis; p < 0.05 was considered statistically significant, and *** p < 0.001 was considered statistically highly significant.

Target SBP/DBP <140/90 mmHg for all hypertensive patients				Target SBP/DBP<130/80 mmHg for hypertensive patients with comorbidity			
Prescription, n (%)	Controlled BP, n (%)	Not controlled BP, n (%)	χ^2^ (p-value)	Prescription, n (%)	Controlled BP, n (%)	Not controlled BP, n (%)	χ^2^ (p-value)
Total adherence, 348 (83.5)	262 (62.8)	86 (20.6)	71.316 (0.000)***	Adherence, 212 (78.5)	95 (36.7)	117 (43.3)	11.784 (0.001)***
Total non-adherence, 69 (16.5)	15 (3.6)	54 (12.9)		Non-adherence, 58 (21.5)	11 (4.1)	47 (17.4)	
Total study population, 417	277 (66.4)	140 (33.6)		Total = 270 (100)	106 (39.3)	164 (60.7)	

## Discussion

To our knowledge, this is the first study evaluating the degree of prescription adherence to treatment guidelines and the rate of BP control in Saudi Arabia. This study demonstrated a 66.3% BP control rate and 83.5% prescription adherence to the hypertension guidelines. The rate of BP control is directly linked to prescription adherence. When clinicians adhere to the hypertension guidelines, BP control is good, and CVD is lowered. The present results are consistent with previous studies conducted in Brazil [5] and Portugal [6].

The primary outcomes of our study were (1) the type of antihypertensive therapy and intensity of BP control depended on the patient’s age; young patients were less aggressively treated (mostly with monotherapy) compared to the older age group. (2) Treatment intensity increased for patients with comorbidity and treatment duration of hypertension. (3) Rate of BP control was linked to a percentage of adherence to the treatment guidelines; non-adherence to treatment showed poor BP control. Treatment intensity (as indicated by polytherapy) increased with age, comorbidities, and duration of hypertension. The broad armamentarium of drug treatment options was applied preferably at a later age and in patients with comorbidities. Our study supports a previous study conducted in Germany [7].

Regarding overall drug utilization, our study revealed that RAS blockers (ACEIs/ARBs) and DHP-CCBs were the most preferred antihypertensive classes, supporting previous hypertension studies conducted in South Korea [11], India [12], and Saudi Arabia [16]. In contrast, TZD-type diuretics were the most prescribed antihypertensive drugs among Portuguese adults [6], possibly due to racial differences between White and Black populations [9]. Notably, in the 2020 ISH Global Hypertension Practice Guidelines, β-blockers were not a part of the first-line therapy, as reflected in our drug utilization study. Regarding the number of medications used, we demonstrated that monotherapy and dual therapy strategies were implemented frequently, aligning with the previous hypertension studies conducted in South Korea [11], India [12], and Saudi Arabia [16].

According to the 2020 ISH Global Hypertension Practice Guidelines, monotherapy should be considered in low-risk grade 1 hypertension, elderly (≥80 years), or frail patients [8]. Our study observed that most monotherapies prescribed to low-risk, grade 1 hypertensive patients supported the hypertension treatment guidelines [8,9] and hypertension studies conducted in Germany [7] and Bahrain [13]. Polytherapy from different classes should be considered for high-risk (DM, CVD, stroke, and CKD) grade 1 hypertension, grade 2 hypertension, and resistant hypertension [8]. Polytherapy is five times more effective than increasing the dose of a single drug [10]. Furthermore, a recent report demonstrated that Saudi physicians primarily prescribed polytherapy of antihypertensive drugs in high-risk grade I and grade II hypertension in adherence to the treatment guidelines [8,9]. The international scenario also suggests a comparable prescription pattern, as reported in studies conducted in Brazil [5], Germany [7], and Bahrain [13].

Comorbidities such as DM, IHD, HF, CKD, stroke, and metabolic syndrome may affect therapeutic choices of antihypertensive drugs [8,9]. Our study demonstrated that almost half of the study population had DM. The co-occurrence of hypertension and DM increases cardiovascular morbidity and mortality [17]. Therefore, strict control of BP in patients with diabetes is crucial. The BP target for patients with DM should be lower (SBP/DBP <130/80 mmHg) [8]. Our study indicated that RAS blockers are the most preferred choice for patients with diabetes, suggesting alignment with the treatment guidelines [8,9] and similar to the results of previous hypertension studies conducted in Portugal [6], north India [18], and Turkey [19]. Hypertensive diabetics are mostly treated with polytherapy of antihypertensive drugs, which is significantly higher than in patients with hypertension alone (53.2% versus 36.7%) and consistent with studies reported by Dhanaraj et al. [18] and Kahya Eren et al. [19].

Regarding drug utilization in DLP patients, our study revealed that the most preferred antihypertensive drugs in dyslipidemia are RAS blockers (79.4%) and DHP-CCBs (58.8%), aligning with the hypertension guidelines and consistent with a previous study in Germany reported by Pittrow et al. [7]. In a few cases of dyslipidemia and CKD, β-blockers were used as dual and triple therapy without compelling indications, considered non-adherence to the treatment guidelines [8,9]. Most antihypertensive guidelines and a hypertension study reported by Dhakam et al. [20] reported that non-vasodilating β-blockers (bisoprolol, metoprolol, atenolol, and propranolol) might aggravate metabolic disorders. Conversely, the newer vasodilating β-blockers (carvedilol, labetalol, and nebivolol) have demonstrated a neutral or favorable metabolic profile. Therefore, the Ministry of Health and physicians of Saudi Arabia urged to revise the clinical use of β-blockers, especially in hypertension patients with metabolic disorders.

According to the hypertension guidelines, using RAS blockers is recommended as the first line in CKD patients because they reduce albuminuria in addition to BP control. In this study, physicians mainly used RAS blockers, revealing adherence to the hypertension guidelines [8,9] and consistent with previous hypertension studies reported by Kim et al. [11] and Pittrow et al. [7].

As first-line therapy, hypertensive patients with IHD should be treated with RAS blockers and β-blockers for compelling indications (previous myocardial infarction and HF with reduced ejection fraction) [8]. Our results demonstrate that RAS blockers and β-blockers are the most preferred antihypertensives among IHD patients, revealing adherence to the hypertension guidelines and consistent with previous hypertension studies conducted in Turkey by Kahya Eren et al. [19] and Norway by Pedersen et al. [21]. The treatment guidelines recommended RAS blockers, DHP-CCBs, and TZD diuretics as first-line antihypertensive agents to prevent recurrent strokes [9]. However, few studies have demonstrated that RAS blockers are less effective than TZD/TZD-like diuretics and DHP-CCBs in lowering BP and preventing stroke [22,23]. DHP-CCBs are a good choice for initial therapy when TZD diuretics are not tolerated [22,23]. In our study, the most preferred choice among stroke patients were RAS blockers (92.3%) and DHP-CCBs (69.2%). Our results align with the hypertension guidelines [8,9] and the results of a previous study conducted in Jordan [24]. RAS blockers, β-blockers, and diuretics improve clinical outcomes in patients with HF [9]. In this study, diuretics and RAS blockers were equally the most preferred choice among CHF patients. However, our results are inconsistent with a previous hypertension study conducted in South Korea [7], where β-blockers were the most preferred choice.

Treatment of elevated BP in advanced age (≥65 years) is challenging due to a high degree of heterogeneity in comorbidity, polypharmacy, and frailty [8]. Our study results revealed that RAS blockers (73.6%) were the most preferred antihypertensives, followed by DHP-CCBs (69.2%) in advance age hypertensive patients. However, previous hypertension studies conducted in Malaysia [25] and the United Kingdom [26] reported that DHP-CCBs were the most preferred choice. Furthermore, of the total, 70.3% of the elderly used polytherapy. The higher use of polytherapy may be due to comorbidities and a longer duration of hypertension in the elderly age group. The treatment guidelines recommend a single drug therapy in low-risk, very old hypertensive patients (age ≥80 years) because intensive BP control increases the risk of acute kidney injury [8]. However, several prescriptions in this study demonstrated that physicians prescribed polytherapy to very old patients (age ≥80 years) and low-risk grade 1 hypertensive patients, revealing non-adherence to the treatment guidelines [8,9]. Age is a compelling indicator for the selection of diuretics [27], and indeed they were prescribed more frequently in elders; however, to a smaller extent than RAS blockers and DHP-CCBs. As combination therapy, diuretics are mainly used with DHP-CCBs and RAS blockers and less likely with β-blockers. The present results support previous studies reported by Sommerauer et al. [27] and Spinar et al. [28].

We highlighted non-adherence to monotherapy, combination therapy, and elderly patient treatment. According to the treatment guidelines, a combination of ACEIs, ARBs, and direct renin inhibitors is not recommended due to the greater risk of hyperkalemia and hypotension and the lack of demonstrated benefit in cardiology trials [29] and diabetic nephropathy trials [30]. Some prescriptions are prescribed with ACEIs as monotherapy in elderly patients (≥75 years), disclosing non-adherence to the treatment guidelines [10]. For elderly hypertensive patients (≥75 years), DHP-CCBs or TZD/TZD-like diuretics should be used instead of ACEI/ARBs due to the risk of hyperkalemia and increased creatinine levels [8]. Some triple therapy was prescribed without TZD/TZD-like diuretics, revealing non-adherence to the treatment guidelines [8,9]. The guidelines recommend that TZD/TZD-like diuretics be added as antihypertensive agents when three or more combination therapies are used [8,9]. Physicians prescribed furosemide without justifiable associated edematous conditions (HF, CKD, and liver failure), which could have been an incorrect indication [5].

Study limitations

Our study result has certain limitations. First, because of the retrospective, cross-sectional design, there was no assessment of whether the current therapy was the initial one or whether it replaced or amended the original therapy. Second, the study was conducted in a single general hospital, and thus the results cannot be generalized to the whole population. Third, only outpatients were included in the study. Further, some information regarding side effects, contraindications, and other comorbidities such as electrolyte abnormalities, gout, bronchial asthma, chronic obstructive pulmonary disease, and secondary hypertension-endocrine causes were not evaluated and might have played a role in drug choice.

## Conclusions

Our study evaluates the prescription pattern in an OPD of a general hospital in Saudi Arabia and its alignment with the 2020 ISH Global Hypertension Practice Guidelines. The current findings indicate that the pattern of prescription is in line with hypertension guidelines and other published hypertension studies. However, considerable scope exists for improvement in rational drug utilization and rate of BP control, particularly in high-risk patients, such as those with DM, DLP, IHD, HF, and CKD, and the elderly. Therefore, treatment guidelines should be followed by clinicians to meet blood pressure goals and reduce cardiovascular events among the Saudi population.
